# Identification of plant microRNAs using convolutional neural network

**DOI:** 10.3389/fpls.2024.1330854

**Published:** 2024-03-19

**Authors:** Yun Zhang, Jianghua Huang, Feixiang Xie, Qian Huang, Hongguan Jiao, Wenbo Cheng

**Affiliations:** College of Information Engineering, Guizhou University of Traditional Chinese Medicine, Guiyang, Guizhou, China

**Keywords:** deep learning, plant, microRNA, Java, SRICATs

## Abstract

MicroRNAs (miRNAs) are of significance in tuning and buffering gene expression. Despite abundant analysis tools that have been developed in the last two decades, plant miRNA identification from next-generation sequencing (NGS) data remains challenging. Here, we show that we can train a convolutional neural network to accurately identify plant miRNAs from NGS data. Based on our methods, we also present a user-friendly pure Java-based software package called Small RNA-related Intelligent and Convenient Analysis Tools (SRICATs). SRICATs encompasses all the necessary steps for plant miRNA analysis. Our results indicate that SRICATs outperforms currently popular software tools on the test data from five plant species. For non-commercial users, SRICATs is freely available at https://sourceforge.net/projects/sricats.

## 1 Introduction

MicroRNAs (miRNAs) are 20–24 nucleotide non-coding RNAs that perform important roles in a wide range of critical cellular processes in animals, plants, and viruses (Zhang et al., 2011). In plants, miRNAs are produced by Dicer-catalyzed excision from stem-loop precursors transcribed from miRNA genes. They are found amid a maelstrom of RNA species called small interfering RNAs (siRNAs), which are processed by Dicer proteins from double-stranded RNA (dsRNA) precursors. The majority of miRNAs are able to negatively regulate gene expression *via* direct RNA-induced silencing complex (RISC) binding to target mRNAs to cause transcript degradation or translational repression, while small fractions have developed specific properties that regulate other silencing pathways (Li and Yu, 2021). Since they are crucial in a number of physiological and developmental processes, miRNAs are regarded as important candidates for bioengineering to improve crop yield and food security (Wai et al., 2017; Wang et al., 2021; He et al., 2022; Su et al., 2023). Additionally, over the last decades, some studies suggest that they may be able to inhibit severe acute respiratory syndrome coronavirus 2 and other viruses such as influenza A viruses, varicella-zoster virus, and enterovirus 71 (Zhou et al., 2015; Li et al., 2018; Huang et al., 2019; Zhou et al., 2020), thus significantly increasing the need for identifying them in many laboratories.

Recent advances in next-generation sequencing (NGS) technology have facilitated the analysis of large plant small RNA-seq datasets. However, accurately identifying miRNAs remains a challenging bioinformatics task. Using features of miRNA biogenesis, several computational tools, including PsRobot (Wu et al., 2012), ShortStack (Axtell, 2013a), miRPlant (An et al., 2014), miRDeep-P (Yang and Li, 2011), miRDeep-P2 (Kuang et al., 2019), miR-PREFeR (Lei and Sun, 2014), miRA (Evers et al., 2015), miReNA (Mathelier and Carbone, 2010), and UEA sRNA workbench (Stocks et al., 2018), have been developed for identifying plant miRNAs from NGS datasets. The presence of hairpin structure has been considered the key criterion for the identification of miRNA precursors (Meyers et al., 2008). However, in plants, a majority of small RNAs are derived from the post-transcriptional processing of RNA precursors, and many siRNA precursors in them can also be folded into hairpin-like structures (Axtell, 2013b). Moreover, most of the plant miRNA precursors are 100–200 bp long, which is much longer than those in animals, and the complex secondary structure of these precursors makes it more difficult to identify accurately. This is particularly true for many plant miRNAs that have relatively large loops, which can fold into several small bifurcate structures. As a result, these tools often generate a significant number of false-positive or false-negative candidates (Axtell and Meyers, 2018).

Machine learning is a field of study that empowers computers to learn without explicit programming. In recent years, deep learning, a branch of machine learning, has emerged as a powerful tool for many bioinformatics tasks (Eraslan et al., 2019). Deep learning has its beginnings in neural networks, which were a computational model sharing some properties with the animal brain. An important breakthrough was made in 2006 when Hinton et al. showed that a deep feed-forward neural network could be pretrained using a stack of restricted Boltzmann machine (RBM), followed by supervised fine-tuning using back-propagation algorithm (Hinton and Salakhutdinov, 2006). Recent advances in deep learning fields, particularly convolutional neural networks (CNNs), have become state-of-the-art for tasks like image recognition and other challenging applications (Silver et al., 2016; Fersht, 2021). CNNs are inspired by the visual cortex of the brain and were first popularized by LeCun (LeCun et al., 1998, 2015). By utilizing a stack of multiple processing layers to represent features of data, CNNs allow automatic learning and extracting features from graph-structured data. CNNs avoid biased *a priori* definitions of features and tend to be most useful when there are some structures in the input data. Plant small RNA precursor data contain a wealth of structural information regarding miRNA and siRNA precursors. Therefore, these datasets are well-suited for analysis using CNNs, which can effectively discover local patterns within the data.

In this study, we demonstrate the successful training of a CNN for the accurate identification of plant miRNAs. Additionally, we introduce Small RNA-related Intelligent and Convenient Analysis Tools (SRICATs), a freely available Java-based package that utilizes our method to identify plant miRNAs from NGS datasets. Our results show that SRICATs has better performance in identifying plant miRNAs than other tools. Moreover, many existing plant small RNA analysis tools are typically command-line driven and often require the installation of numerous third-party software packages, such as genomic mapping and RNA secondary structure prediction tools. In our package, we have integrated rewritten Java-based programs of all these third-party tools into a Java library, seamlessly linked to our program. We have also developed a user-friendly graphical interface using JavaFX and additional Java-based programs for various miRNA analysis tasks. Ultimately, we have consolidated all these programs into a pure Java-based package, providing users with a high-performance and easy-to-use plant miRNA analysis tool.

## 2 Materials and methods

### 2.1 Datasets

The training data for our study consisted of datasets from two model plants, *Oryza sativa* and *Arabidopsis thaliana*. The genome sequences of *O. sativa* ssp. *japonica* and *A. thaliana* were collected from release 7.0 of the Rice Genome Annotation Project Database (https://rice.plantbiology.msu.edu) and the release 10 of the Arabidopsis Information Resource database (https://www.arabidopsis.org/), respectively. Small RNA datasets of different tissues of *O. sativa* ssp. *japonica* (GSM2883136, GSM2883137, GSM2883138, GSM2883139, GSM2883140, GSM2883141, GSM3030846, GSM3030847, GSM3030848, GSM3030849, GSM3030850, and GSM3030851) and *A. thaliana* (GSM2094927 and GSM2412287) were downloaded from the National Center for Biotechnology Information database (https://www.ncbi.nlm.nih.gov) (see **Supplementary File S1**).

### 2.2 Data processing

**Figure 1:**
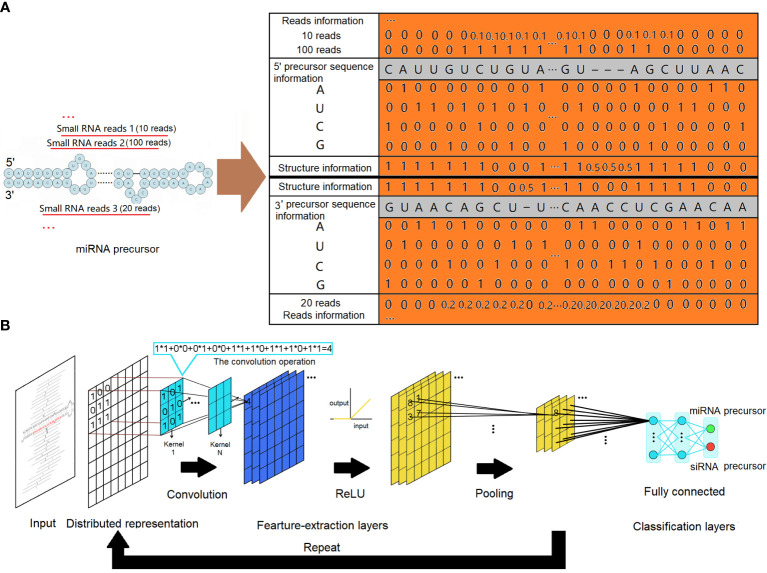
MiRNA precursor structure recognition with a convolutional neural network. **(A)** Distributed representations for a precursor. The sequence information is represented by one-hot codes of the four nucleotides of RNA. The structure information is represented by digital codes: 1 represents that the corresponding base is paired, 0 represents that the corresponding base is unpaired, and 0.5 represents a gap. Normalized digital codes represent reads information. The final matrix data obtained are represented by the orange area. **(B)** The architecture of the convolutional neural network. The network consists of an input layer followed by a certain number of feature-extraction layers and fully connected classification layers. The feature-extraction layers have a generally repeating pattern of the sequence: convolution layer, ReLU layer, and pooling layer. ReLU, rectified linear unit.

To ensure the accuracy of our analysis, small RNA reads falling outside the miRNA length range (20–24 nucleotides) were excluded. The remaining reads were then mapped onto the corresponding genome. From the matched regions, precursor sequences were extracted by including various lengths of upstream and downstream sequences. The secondary structures of the extracted precursor sequences were evaluated using the miRPlant program, and precursors with hairpin secondary structures were identified (An et al., 2013, 2014). MiRNA precursors were then identified from these precursors using a strict set of criteria, following the recommendations of Meyers et al (Axtell and Meyers, 2018). The input to our CNNs was a distributed representation of sequence and structure information of all precursors with hairpin secondary structures. As depicted in **Figure 1A**, the four nucleotides (“A”, “U”, “C”, and “G”) were encoded using one-hot coding in each column of the matrix. The secondary structures of the precursors were represented using a two-dimensional vector. In this representation, a value of 1 indicated that the corresponding base is paired, a value of 0 indicated that the corresponding base is unpaired, and a value of 0.5 represented a gap. Individual small RNA reads mapped to the precursors were represented based on their positions and expressions. To ensure fair representation, the expressions of these small RNAs were normalized by dividing the number of reads of each small RNA by the number of reads of the small RNA with the maximum expression. This normalization step was necessary to prevent extreme input values from adversely affecting the training process. The CNN is a supervised machine learning method. Given training data in the form of input–output pairs, it trains a model that can best fit the training data. In our case, a dataset of distributed representation of miRNA precursors and other hairpin secondary structure precursors was collected, each labeled with its category as the output of training data. Our data were observed to be imbalanced, with the number of negative samples being approximately three times that of positive samples. This imbalance can mislead the model, causing it to overlearn the majority class and potentially affect its performance. To address this issue, we applied resampling techniques by oversampling the minority class in the training data, resulting in a balanced training dataset.

### 2.3 CNN architecture

Regardless of the specific use case, the architecture of CNNs typically comprises an input layer, followed by a series of feature-extraction layers and connected layers. In this architecture, the initial feature-extraction layers are responsible for capturing low-complexity fundamental features. As the data flow through subsequent feature-extraction layers, more complex features are formed through intricate combinations of the low-complexity features. This hierarchical process allows the network to progressively learn and represent increasingly sophisticated patterns and structures in the input data.

The feature-extraction layers have a general repeating pattern of the sequence: convolution layer, rectified linear unit (ReLU) layer, and pooling layer. A convolution is defined as a mathematical operation describing a rule for how to merge two sets of information. It defines a bridge between the space/time domain and the frequency domain through the use of Fourier transforms. The convolution operation, shown in **Figure 1B**, is known as the feature detector of a CNN. It takes input, applies a convolution kernel (or filter), and gives us a feature map as output. The convolution kernels can be thought of as local feature extractors, as their output only depends on pixels in close spatial proximity. Convolution layers make use of a series of convolution kernels of a particular size and are optimized to find the major characteristics of the input matrices being subject to a sophisticated training process. In a mathematical sense, convolutions are linear operations. For CNNs, all convolution operation outputs are commonly transformed by the ReLU activation function for introducing non-linearity to have richer representational power than a simple linear model. The ReLU activation function applies a max(0, x) operation to the input data. It also can avoid the gradient vanishing problem and has better convergence performance. In general, the convolution layer computes its output by performing the convolution operation with a specified number of kernels, and all the outputs are then transformed by the ReLU activation function:


$$X_{i,k}^{m,l}=ReLU\left(\sum_{x=1}^{s}\sum_{y=1}^{n}W_{x,y}^{k,l-1}X_{i+x,y}^{m,l-1}\right)$$

In the given equation, *X* represents the input, *m* denotes the index of the miRNA precursor, *l* represents the index of the convolution layer, *i* corresponds to the index of the output position, and *k* signifies the index of the convolution kernels. Each convolution kernel, denoted as *W^k^* ^,*l*^, is a weight matrix of size *s* × *n* for convolution kernel *k* at layer *l*. Here, *s* represents the window size, and *n* indicates the number of input channels.

After the convolution and ReLU layers, a pooling layer is often used to reduce the spatial size of the data representation and control overfitting. Additionally, it facilitates motif translation invariance, ensuring that the desired motif can be captured regardless of its location:


$$X_{i,k}^{m,l}=pool\left(\left\{X_{is,k}^{m,l-1},X_{is+1,k}^{m,l-1}\ldots X_{is+s-1,k}^{m,l-1}\right\}\right)$$

In the given context, *X^m^* ^,*l*^ represents the input of miRNA precursor *m* from the preceding convolution layer *l* − 1, *i* denotes the index of the output position, *k* signifies the index of the kernel, and *s* represents the pooling window size. Within such a layer, subsets of each filtered matrix are pooled according to their mean or maximum values. For our CNN, we used the max() operation. The max() operation computes the maximum value in a window of spatially adjacent convolution layer outputs for each kernel, with a step size equal to the size of the pooling window. Finally, fully connected layers aggregate the weights from the previous layers to determine a precise combination of features that contribute to a specific target output. In our architecture, we employed two fully connected layers, followed by a softmax layer at the end. The softmax layer computes class scores, which serve as the network’s output.

### 2.4 Training of CNNs

Our CNNs were trained using DeepLearning4J. The training data were used to train the CNN model by minimizing the loss function. The loss function calculates the error at the target layer between the actual outputs associated with the training input and the desired outputs generated from the network. Looking for the ideal state of the network is equivalent to finding the parameters that could minimize errors. Thus, the loss function helps reframe training neural networks as an optimization problem that can be approximated and solved with iterative optimization algorithms like gradient descent. A method called the error backpropagation algorithm is used for reducing errors in CNNs. We can consider backpropagation to be doing gradient descent in weight space where the gradient is on the error surface. The amount of the weights that are changed with each iteration is known as the learning rate. During the backward pass for each layer, the errors are used in a feedback mechanism in a layer-by-layer fashion to update the parameters until a satisfactory level of decision accuracy is achieved at the target layer.

To reduce overfitting and train robust features, drop-out is used after each of the hidden layers. Dropout regularizes the neural network by stochastically removing some neurons and their connections from the CNNs at training time. This has the effect of preventing coadaptation between neurons, which may not generalize well outside of the training data.

## 3 Results

### 3.1 Determination of the CNN architecture and hyperparameters

To evaluate the performance of the model, five-fold cross-validation was employed. In this approach, the training dataset was randomly divided into five equal-sized subsets. Four subsets were used for training, while one subset served as the test group. The model was trained on the training group and tested on the test group. This process was repeated five times, and the five results were averaged to produce a single estimation. Thus, the true-positive (TP) rate, false-positive (FP) rate, true-negative (TN) rate, and false-negative (FN) rate of the model can be evaluated. The TP is defined if the sample is labeled as miRNA and the prediction is also miRNA. The FP is defined if the sample is not labeled as miRNA but the prediction is miRNA. In traditional statistics, it is also known as “type I error”. The TN is defined if the sample is not labeled as miRNA and the prediction is also not miRNA. The FN is defined if the sample is labeled as miRNA but the prediction is not miRNA. In traditional statistics, it is also known as “type II error”. Then the accuracy, precision, recall, and F1-score of the model were calculated to test the performance of our CNNs. The accuracy is the proportion of all predictions that are correct:


Accuracy=(TP+TN)/(TP+FP+FN+TN)

Accuracy represents the degree of closeness between measurements of a quantity and its true value. Precision, in contrast, measures the proportion of positive predictions that are correct:


Precision=TP/(TP+FP)

Precision reflects the consistency of results obtained from repeated measurements under the same conditions. Recall measures the proportion of actual positive observations that are correctly identified:


Recall=TP/(TP+FN)

Recall quantifies how well the model avoids false negatives by capturing how often an input record is correctly classified as the positive class. The F1-score combines both precision and recall into a single score using the harmonic mean:


F1-score=2∗TP/(2∗TP+FP+FN)

In binary classification, the F1-score is commonly used as an overall measure of how well a model performs. During the training of the CNNs, we tested various CNN architectures and hyperparameters (**Supplementary Table S1**). The final model achieved an accuracy of 97.56%, a precision of 95.08%, a recall of 100%, and an F1-score of 97.48% (**Table 1**). Interestingly, we found that the architecture and hyperparameters of this model were very similar to those of LeNet (LeCun et al., 1998). We speculate that LeNet has also undergone various tests to discover this optimal architecture and hyperparameters.

**Table 1:** CNN architectures and hyperparameters to be tuned.

CNN architectures and hyperparameters	Range	Final value
**Number of convolution layers and pooling layers** **Filter size of first convolution layer** **Number of filters in first convolution layer** **Activation function in first convolution layer** **Filter size of second convolution layer** **Number of filters in second convolution layer** **Activation function in second convolution layer** **Stride for filters** **Using padding** **Pooling method** **Filter size of pooling layer** **Number of units in fully connected layer** **Activation function in fully connected layer** **Activation function in output layer** **Regularization technique** **Dropout coefficient** **Optimization algorithm** **Loss function for classification** **Weight initialization strategies**	**2, 3** 2 * 2, 3 * 3, 4 * 4, 5 * 5 **20, 30, 40, 50, 60, 70, 80, 90,** **100, 300, 500** **ReLU, Identity, Tanh, Sigmoid** 2 * 2, 3 * 3, 4 * 4, 5 * 5 **20, 30, 40, 50, 60, 70, 80, 90,** **100, 300, 500** **ReLU, Identity, Tanh, Sigmoid** 1 * 1, 2 * 2 **Yes, No** **Max pooling, Average pooling** 2 * 2, 3 * 3 **100, 300, 500, 700, 1,000** **ReLU, Identity, Tanh, Sigmoid** **Sigmoid, Softmax** **L1, L2, Dropout** **0.5, 0.6, 0.7, 0.8, 0.9, 1.0** **Adam, AdaGrad, AdaDelta,** **RMSProp, Momentum SGD** **Hinge, Negative log likelihood** **ReLU, Xavier**	**2** 2 * 2 **20** **ReLU** 2 * 2 **60** **ReLU** 1 * 1 **No** **Max pooling** 2 * 2 **500** **Identity** **Softmax** **Dropout** **0.9** **AdaDelta** **Negative log likelihood** **Xavier**

The performance of CNN architectures and hyperparameters tested can be found in **Supplementary Table S1**.

CNN, convolutional neural network; ReLU, rectified linear unit.

### 3.2 A pure Java-based package—SRICATs

**Figure 2:**
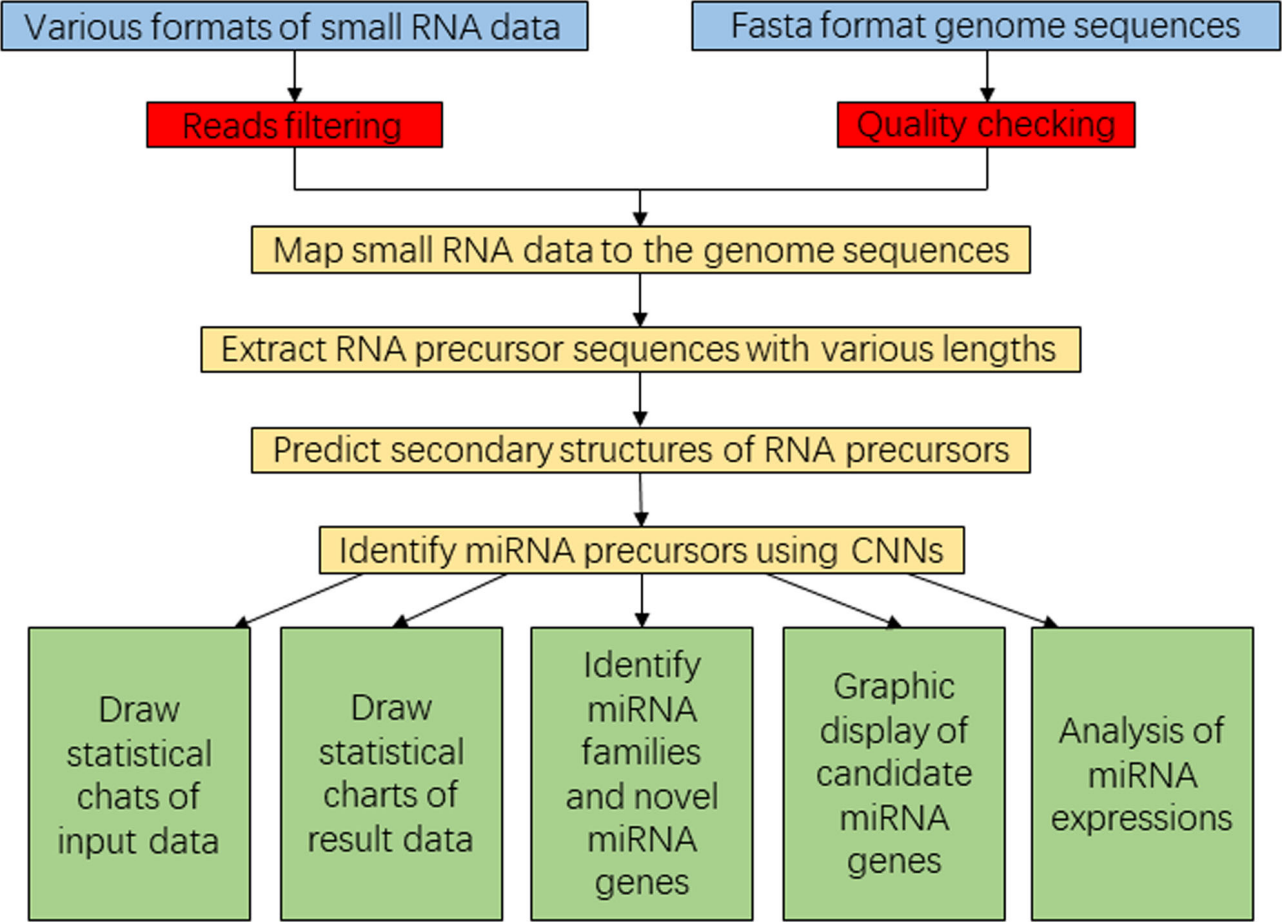
Flowchart diagram representing the SRICATs software package. SRICATs, Small RNA-related Intelligent and Convenient Analysis Tools.

**Figure 3:**
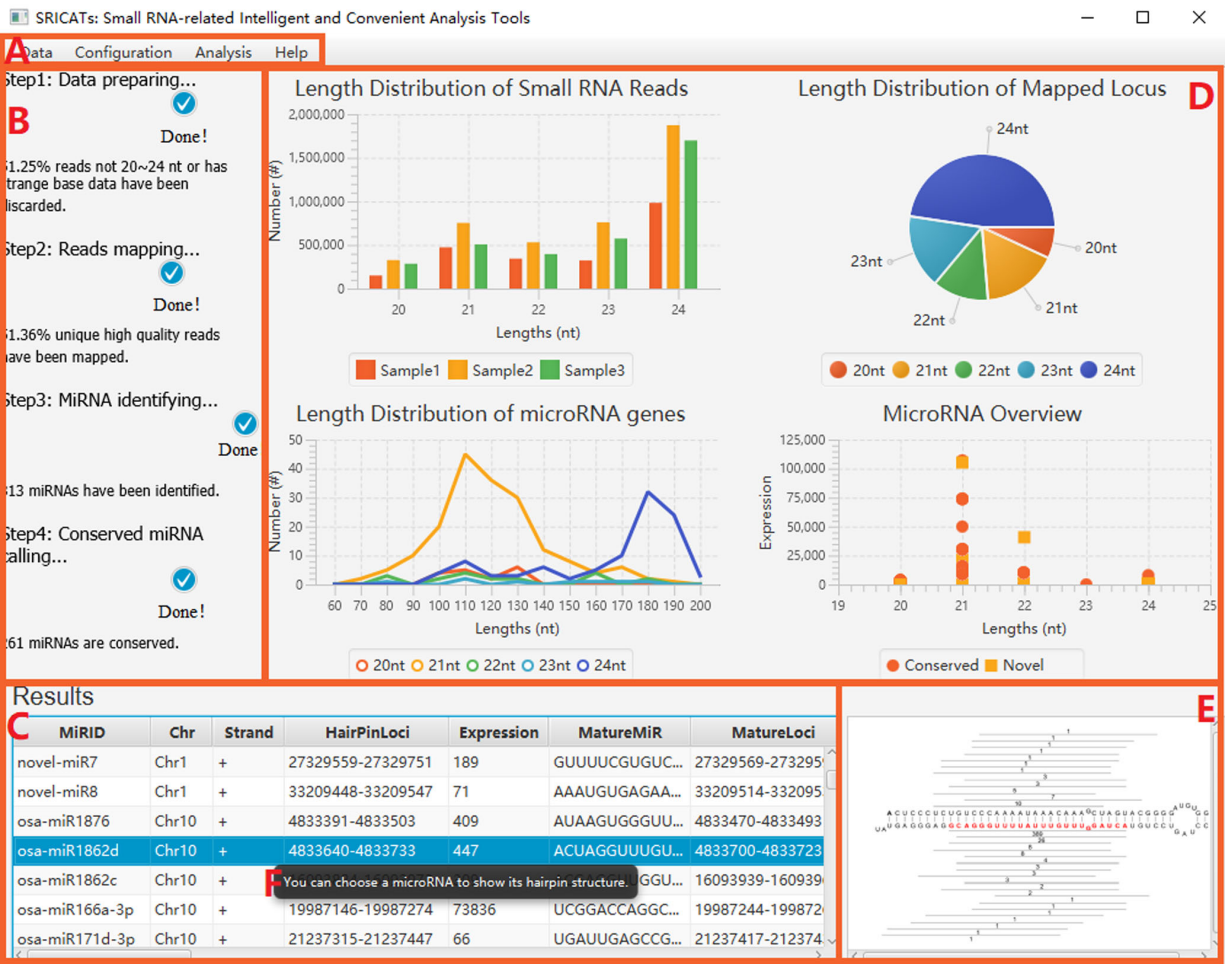
Screenshot of SRICATs. **(A)** Menu bar. **(B)** Progress panel displaying the progress of tasks. **(C)** Results panel showing attributes of annotated miRNAs. **(D)** Statistical chart panel displaying basic features of the uploaded dataset and results. **(E)** Graph panel displaying the secondary structure of selected miRNA precursors. **(F)** Intelligent prompts are displayed when mousing over the corresponding regions. SRICATs, Small RNA-related Intelligent and Convenient Analysis Tools.

To enhance user convenience, we have integrated all our programs into a comprehensive Java-based package called SRICATs. SRICATs supports a variety of file types from raw data to processed data. An overview of the SRICATs program is shown in **Figure 2**. SRICATs first filtered small RNA reads and mapped them onto the corresponding genome. For a given mapped reads, SRICATs gathered sequences in the reference genome flanking the reads and computed their secondary RNA structures using miRPlant, a Java-based miRNA precursor secondary structure calculation tool (An et al., 2013, 2014). Then, the sequence structure data were transformed into distributed representation data, and miRNA precursors were identified using pretrained CNN models. Additionally, SRICATs also provided an all-in-one plant miRNA analysis platform for users. The functions of this platform include i) processing different types of input data and analyzing multiple samples simultaneously, ii) checking the quality of genome data and filtering low-quality small RNA reads, iii) generating statistical charts for input and result data, iv) identifying miRNA families by comparing program outputs to miRBase (Kozomara et al., 2019), v) converting text outputs of secondary structure information of miRNA precursors to a user-friendly visual output, vi) analyzing miRNA expression status, and vii) supporting parameter adjustment and model selection [as plant miRNAs with diverse genomic origins have different structural characteristics (Zhang et al., 2011), we trained different CNN models to meet the needs of various users] (see **Supplementary File S2**). To date, most plant small RNA analysis tools are typically command-line driven and require the installation of multiple third-party software. We developed a user-friendly graphical interface using JavaFX and integrated rewritten Java-based programs of all third-party software into our package. Thus, we provided an easy-to-use, flexible, and robust plant miRNA analysis tool for users (see **Figure 3**).

### 3.3 Comparison of SRICATs with existing plant miRNA identification programs

**Figure 4:**
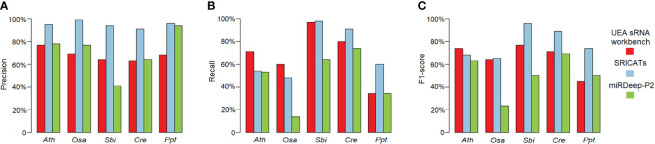
Comparison of SRICATs and two other programs for plant miRNA identification. **(A)** Precision comparison between SRICATs and the other two programs. **(B)** Recall comparison between SRICATs and the other two programs. **(C)** F1-score comparison between SRICATs and the other two programs. Ath, *Arabidopsis thaliana*; Osa, *Oryza sativa*; Sbi, *Sorghum bicolor*; Cre, *Chlamydomonas reinhardtii*; Ppt, *Physcomitrella patens*; SRICATs, Small RNA-related Intelligent and Convenient Analysis Tools.

We conducted a comprehensive comparison of SRICATs with two well-known plant miRNA identification programs: miRDeep-P2 and UEA sRNA workbench. miRDeep-P2 is currently the most widely used program for plant miRNA identification and has demonstrated superior performance compared to other commonly used programs, such as miRDeep-P, miRPlant, miR-PREFeR, miRA, TripletSVM, and miReNA (Kuang et al., 2019; Zhao et al., 2021). UEA sRNA workbench is a Java-based small RNA analysis tool that can also identify plant miRNAs (Stocks et al., 2018). We evaluated the performances of each program on five plant species: *O. sativa*, *A. thaliana*, *Sorghum bicolor*, *Chlamydomonas reinhardtii*, and *Physcomitrella patens*. The small RNA libraries used in our study are listed in **Table 2**. The number of identified miRNAs by SRICATs falls within the range of miRDeep-P2 and UEA sRNA workbench (see **Supplementary Table S2**). We used precision, recall, and F1-score to qualify the results from the programs compared. We did not use accuracy because the true-negative rate in the data that we tested was not known. Based on previously described plant miRNA annotation criteria (exclude secondary stems or large loops in the miRNA/miRNA* duplex; up to five mismatched positions, only three of which are nucleotides in asymmetric bulges) (Axtell and Meyers, 2018), we found that SRICATs has the highest precision in identifying miRNAs in all tested plant species (see **Figure 4A**). We used high-confidence miRNAs from miRBase (version 21) as benchmarks to evaluate false-negative rates. To identify known miRNAs in our results, we compared the candidate miRNA sequences with those of published miRNAs using BLASTN search in miRbase. We regarded sequences with more than 18 matches to currently known miRNAs from all plant species as known miRNAs. The difference between the number of miRNAs in miRbase and the number of known miRNAs in our results can be considered as a false-negative rate approximately. We found that SRICATs also have the highest recall in *S. bicolor*, *C. reinhardtii*, and *P. patens* (see **Figure 4B**) and the highest F1-score in *O. sativa*, *S. bicolor*, *C. reinhardtii*, and *P. patens* (see **Figure 4C**). Overall, SRICATs outperformed other programs in 12 out of 15 tests (80%), indicating its good performance in identifying plant miRNAs.

**Table 2:** Resources for testing SRICATs and other tools.

Species (abb.)	Genome version	sRNA libraries			
		Library ID	File size	Last update date	Number of samples
*Arabidopsis thaliana* (*Ath*)	Version 10	GSE113029	110.6 Mb	2020-7-8	8
*Oryza sativa* (*Osa*)	Version 7	GSE26357	77.6 Mb	2019-5-15	4
*Sorghum bicolor* (*Sbi*)	Version 3	GSM4769351	27.5 Mb	2021-12-31	1
*Chlamydomonas reinhardtii* (*Cre*)	Version 5	GSM803103	4.0 Mb	2013-9-26	1
*Physcomitrella patens* (*Ppt*)	Version 3	GSE44900	67.7 Mb	2019-5-15	10

The details of repository from which we downloaded the datasets can be found in **Supplementary File S1**.

SRICATs, Small RNA-related Intelligent and Convenient Analysis Tools.

## 4 Discussion

In this study, we propose a deep learning-based approach for accurate identification of plant miRNAs. Our findings demonstrate that relying solely on predefined features with plant miRNA precursors is insufficient for precise identification. However, by leveraging CNNs to learn representations from the raw sequence and structure information of plant miRNA precursors, we observe a significant improvement in performance compared to currently popular methods.

To facilitate plant miRNA analysis in a flexible and user-friendly manner, we have developed a pure Java-based software package called SRICATs. Researchers can utilize SRICATs to perform all stages of plant miRNA analysis. We are pleased to offer this software package as an open-source tool, freely available to the academic community at https://sourceforge.net/projects/sricats. The package includes comprehensive documentation with detailed execution instructions (**Supplementary File S2**).

Moving forward, we have plans for expanding the software package. Our ongoing efforts involve incorporating plant siRNA analysis and animal small RNA analysis into SRICATs. Furthermore, we will introduce additional functionalities for small RNA-related analysis and incorporate more deep learning methods. We are committed to continuously enhancing SRICATs to meet the evolving needs of researchers in the field of small RNA analysis.

## Data availability statement

The original contributions presented in the study are included in the article/**Supplementary Material**. Further inquiries can be directed to the corresponding author.

## Author contributions

YZ: Conceptualization, Data curation, Formal Analysis, Funding acquisition, Methodology, Software, Supervision, Visualization, Writing – original draft. JH: Methodology, Project administration, Writing – review & editing. FX: Writing – review & editing. QH: Writing – review & editing. HJ: Writing – review & editing. WC: Writing – review & editing.
