# Supplementary material for: Identification of plant microRNAs using convolutional neural network
Source: Front Plant Sci. 2024 Mar 19;15:1330854. doi: 10.3389/fpls.2024.1330854 (PMC10985208; doi:10.3389/fpls.2024.1330854)
Supplement: Supplementary file 1 [file DataSheet_1.docx]

Supplementary File S1.

Publicly available datasets analyzed in this study can be found here:

Training data sets of genome data:

(1)  *Oryza sativa*:

<https://rice.uga.edu/pub/data/Eukaryotic_Projects/o_sativa/annotation_dbs/pseudomolecules/version_7.0/>

(2)  *Arabidopsis thaliana:*

<https://www.arabidopsis.org/download/index.jsp>

Training data sets of small RNAs:

(1)*Oryza sativa*:

<https://www.ncbi.nlm.nih.gov/biosample/?term=GSM2883136>

<https://www.ncbi.nlm.nih.gov/biosample/?term=GSM2883137>

<https://www.ncbi.nlm.nih.gov/biosample/?term=GSM2883138>

<https://www.ncbi.nlm.nih.gov/biosample/?term=GSM2883139>

<https://www.ncbi.nlm.nih.gov/biosample/?term=GSM2883140>

<https://www.ncbi.nlm.nih.gov/biosample/?term=GSM2883141>

<https://www.ncbi.nlm.nih.gov/biosample/?term=GSM2883146>

<https://www.ncbi.nlm.nih.gov/biosample/?term=GSM2883147>

<https://www.ncbi.nlm.nih.gov/biosample/?term=GSM2883148>

<https://www.ncbi.nlm.nih.gov/biosample/?term=GSM2883149>

<https://www.ncbi.nlm.nih.gov/biosample/?term=GSM2883150>

<https://www.ncbi.nlm.nih.gov/biosample/?term=GSM2883151>

(2)   *Arabidopsis thaliana:*

<https://www.ncbi.nlm.nih.gov/biosample/?term=GSM2094927>

<https://www.ncbi.nlm.nih.gov/biosample/?term=GSM2412287>

Test data sets of genome data:

*(1)*   *Arabidopsis thaliana:*

<https://www.arabidopsis.org/download/index.jsp>

*(2)*   *Oryza sativa*:

<https://rice.uga.edu/pub/data/Eukaryotic_Projects/o_sativa/annotation_dbs/pseudomolecules/version_7.0/>

*(3)*   *Sorghum bicolor:*

[https:// www.ncbi.nlm.nih.gov/datasets/genome/GCF_000003195.3/](https://www.ncbi.nlm.nih.gov/datasets/taxonomy/4558/)

*(4)*   *Chlamydomonas reinhardtii:*

[www.ncbi.nlm.nih.gov/datasets/genome/GCF_000002595.2/](http://www.ncbi.nlm.nih.gov/datasets/genome/GCF_000002595.2/)

*(5)*   *Physcomitrella patens:*

[https:// www.ncbi.nlm.nih.gov/datasets/genome/GCF_000002425.4/](https://www.ncbi.nlm.nih.gov/datasets/taxonomy/3218/)

 Test data sets of small RNAs:

*(1)*   *Arabidopsis thaliana:*

<https://www.ncbi.nlm.nih.gov/bioproject/?term=GSE113029>

*(2)*   *Oryza sativa:*

<https://www.ncbi.nlm.nih.gov/bioproject/?term=GSE26357>

*(3)*   *Sorghum bicolor:*

<https://www.ncbi.nlm.nih.gov/biosample/?term=GSM4769351>

*(4)*   *Chlamydomonas reinhardtii:*

<https://www.ncbi.nlm.nih.gov/biosample/?term=GSM803103>

*(5)*   *Physcomitrella patens:*

<https://www.ncbi.nlm.nih.gov/biosample/?term=GSE44900>

High confidence miRNAs from miRbase (version 21):

<https://mirbase.org/download/PREVIOUS_RELEASES/>
