# Supplementary material for: Identification of plant microRNAs using convolutional neural network
Source: Front Plant Sci. 2024 Mar 19;15:1330854. doi: 10.3389/fpls.2024.1330854 (PMC10985208; doi:10.3389/fpls.2024.1330854)
Supplement: Supplementary file 2 [file DataSheet_2.docx]

Supplementary File S2:

User Guide

SRICATs:

Small RNA-related Intelligent and Convenient Analysis Tools

Version 1.0 (October 2023)

Yun Zhang et al.

Contents

1. Introduction…………………………………………………1

2. License……………………………………………………...2

3. Installation…………………………………………………..2

4. Usage…..………………………………………….………..3

4.1. Read data…………………………………………...3

4.2. Configuration………………………………………..5

4.3. Identify miRNAs..…………………………………...9

4.4. Identify miRNA families……………………..……10

4.5. Help………………………………………………...10

4.6. Save results………………………………………..11

4.7. Exit………………………………………………….11

5. Example…………………………………...………………12

6. Addresses and Contacts………………………………...12

7. References………………………………………………..12

1. **Introduction**

MicroRNAs (miRNAs) are 20~24 nucleotide noncoding RNAs that perform important roles in a wide range of critical cellular processes in animals, plants and viruses (1). Recent advances in next-generation sequencing (NGS) technology have enabled the analysis of large plant small RNA-seq datasets, but the bioinformational task of accurately identifying the miRNAs still remain challenging (2-4).

In recent years, deep leaning has emerged as powerful tool for many bioinformatical tasks (5). To identify plant miRNA accurately, we developed SRICATs (Small RNA-related Intelligent and Convenient Analysis Tools) version 1, a Java-based freely available package, using convolutional neural networks. The performance of our program increases significantly compared to currently popular programs of plant miRNA identification. We also developed a user-friendly graphical interface using JavaFX and some Java-based programs for miRNA analysis tasks such as small RNA reads filtering and miRNA family identification etc. We integrated all programs into a pure Java-based package and provided a high performance and easy-to-use plant miRNA analysis tools for users.

In the near future, the software package will be expanded. The extensions of we are working on include plant siRNA analysis and animal small RNA analysis. Additionally, more functions for small RNA-related analysis and more deep-learning methods will also be added.

1. **License**

SRICATs.

Copyright (C) 2023 Yun Zhang.

This program is free software: you can redistribute it and/or modify it under the terms of the GNU General Public License as published by the Free Software Foundation, either version 3 of the License, or (at your option) any later version.

This program is distributed in the hope that it will be useful, but WITHOUT ANY WARRANTY; without even the implied warranty of MERCHANTABILITY or FITNESS FOR A PARTICULAR PURPOSE. See the GNU General Public License for more details.

You should have received a copy of the GNU General Public License along with this program. If not, see <https://www.gnu.org/licenses/>.

1. **Installation**

In our package, we integrated rewritten Java-based programs of all third-party tools into a Java library. Thus, there is no real installation of SRICATs.

To run the program from the command line, just need install JDK 8 (We tested the program using jdk1.8.0_241. If the program does not work properly, it is probably because of the jdk versions are incompatible, especially for JavaFX. You can use jdk1.8.0_241, then the program will work well.) and simply download the program from https://sourceforge.net/projects[/sricats](https://github.com/BioinformaticsOfGuizhouUniversityOfTCM/SRICATs). For Windows user, type the following: Java -jar SRICATs.jar

For Linux user, type the following: Java -jar SRICATs_forLinux.jar

For Mac user, type the following: Java -jar SRICATs_forMac.jar

(Because some programs involving matrix operation in deep learning packages are large and different operating system use different program, we customized different runnable packages for each operating system. SRICATs.jar has been tested many times under various conditions. Thus, we recommend that SRICATs.jar should be the first choice for plant miRNA identification task.)

You can add the option -Xms to allocate more memory if you plan to produce a huge database.

**4. Usage**

**4.1. Read data**

To start the whole analysis process of the program, you must read genome data and small RNA data firstly.

To read genome data, you can click “Data” menu and choose “Read genome data from Fasta file” (see Figure 1), then choose genome data file of Fasta format.


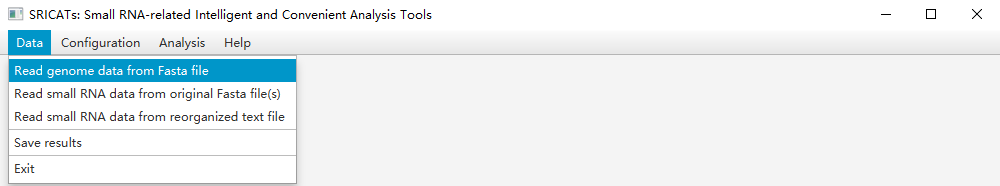


**Figure 1. Read genome data from Fasta file.**

To read small RNA data from original small RNA data file(s) of Fasta format (SRICATs can analyze multiple samples at once), you can click “Data” menu and choose “Read small RNA data from original Fasta file(s)” (see Figure 2).


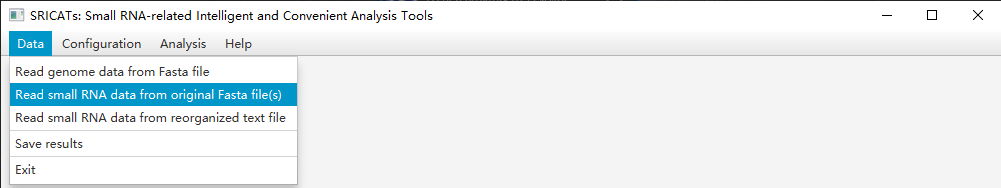


**Figure 2. Read small RNA data from original Fasta file(s).**

Then choose small RNA data file(s) of Fasta format (see Figure 3).


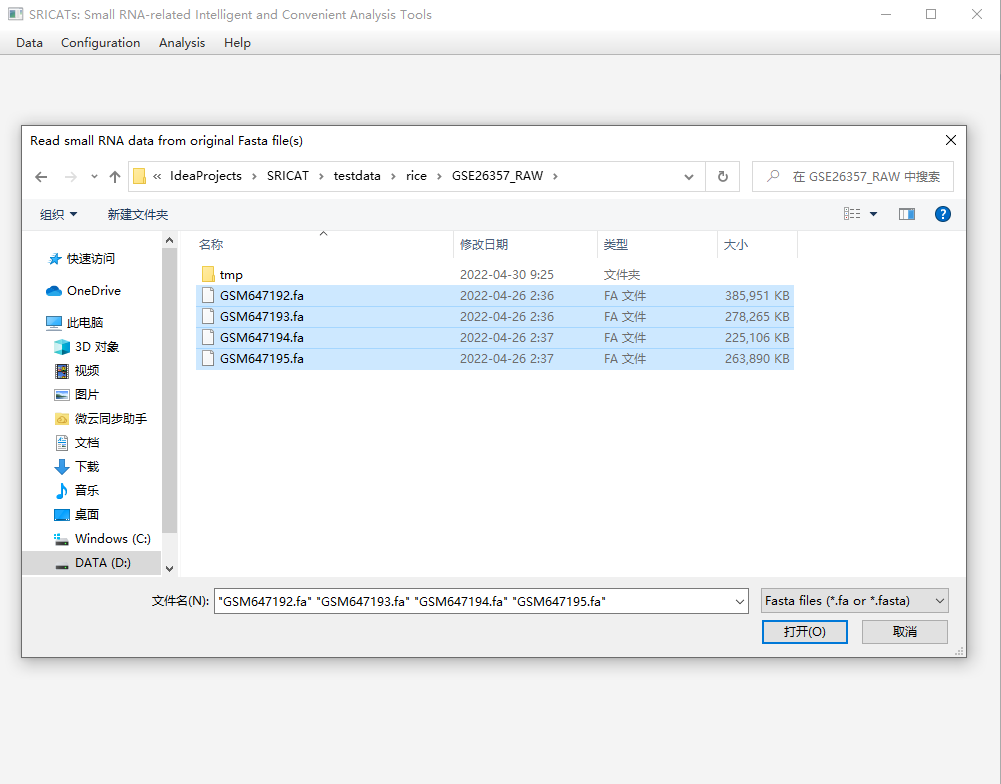


**Figure 3. Read small RNA data from original Fasta file(s).**

Or if you have reorganized small RNA file, for example:

AAAAATTTTTTTTTTTTGGGUUUUUU 3

AATAATTTGGTTTTTTGGGUUUAUU 9

Or

AAAAATTTTGGUTTTTTGGGGUUUUU 3_7_5

ATAAATTTTGGUTTUTTGGGGUUAAAU 50_6_4

The number represent reads number of this sequence. The first example is for one sample. The second example is for three samples. The sequence data can only contain ‘A’, ‘T’, ‘C’, ‘G’, ‘U’, ‘a’, ‘t’, ‘c’, ‘g’ or ‘u’.

You can click “Data” menu and choose “Read small RNA data from reorganized text file” (see Figure 4).


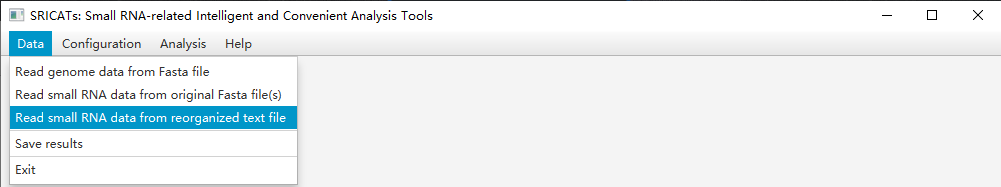


**Figure 4. Read small RNA data from reorganized text file.**

Then choose small RNA data file (see Figure 5).


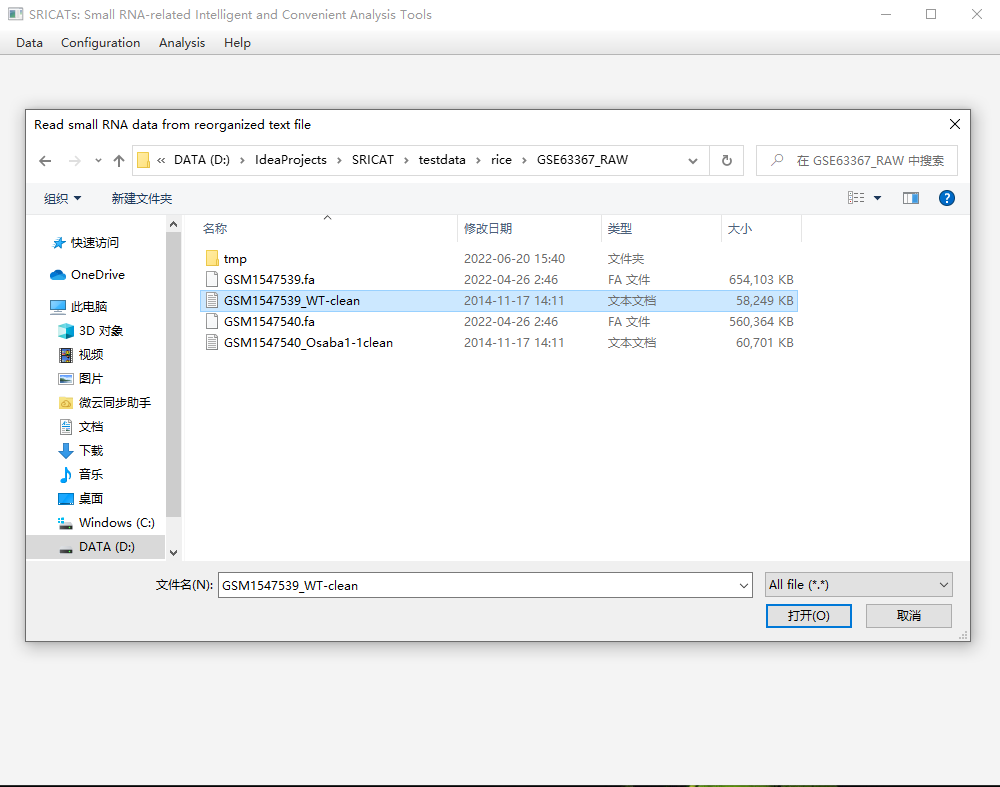


**Figure 5. Read small RNA data from reorganized text file.**

**4.2. Configuration**

Before analysis, you can click “Configuration” menu and choose “Plant small data analysis”, then you can set parameters for plant small RNA data analysis (see Figure 6).


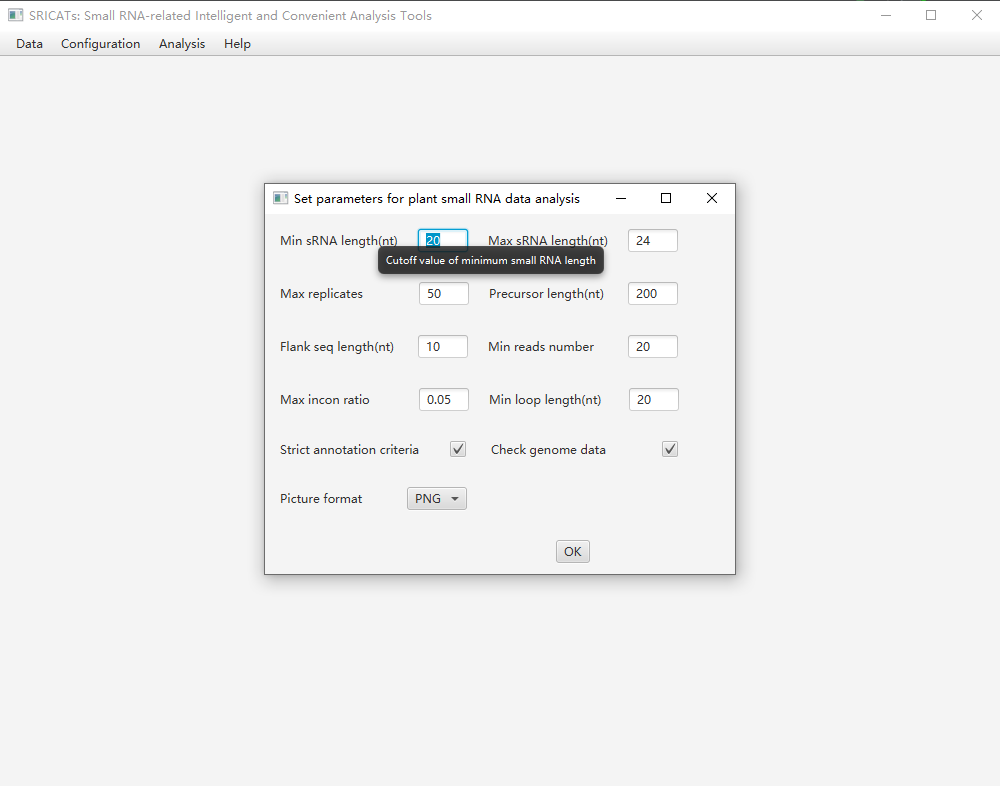


**Figure 6. Set parameters for plant small RNA data analysis.**

Parameters include:

1. Min sRNA length(nt):

Cutoff value of minimum small RNA length (Default value: 20).

1. Max sRNA length(nt):

Cutoff value of maximum small RNA length (Default value: 24).

1. Max replicates:

Maximum replicates of small RNA locus (Default value: 50).

1. Precursor length(nt):

The maximum length of miRNA precursor is less than this value plus maximum sRNA length. Setting this value too large may result in too many wrong results (Default value: 200).

1. Flank seq length(nt):

Length from the end of precursor to mature miRNA (Default value: 10).

1. Min reads number:

Minimum miRNA sequence reads number (Default value: 20).

1. Max incon ratio:

Incon ratio = Number of inconsistent reads aligned to precursor / (number of inconsistent reads + number of consistent reads). A read is in consistence if it aligns with the potential mature miRNA or miRNA* sequences, allowing the read to stretch two nucleotides beyond the expected position in the 5' end or up to five nucleotides in the 3' end. In identifying miRNAs, SRICATs requires less than 5% (max incon ratio option in configuration menu by default) of reads falling out of the predicted miRNA and miRNA* sequence. This rule is used to add robustness to the detection of fuzzy endonuclease processing (Default value: 0.05).

1. Min loop length (nt):

Minimum loop length of miRNA hairpin structure (Default value: 200).

1. Strict annotation model:

As plant miRNAs with diverse genomic origins have different structural characteristics (6), we trained different CNN models to meet the needs of various users. Strict miRNA annotation model, which is suitable for annotating conserved miRNAs, require standard hairpin structure (Refer to the article: Axtell, M. J. & Meyers, B. C. Revisiting Criteria for Plant MicroRNA Annotation in the Era of Big Data. Plant Cell 30, 272–284, https://doi.org/10.1105/tpc.17.00851 (2018). The following criteria are especially important: [1] exclude secondary stems or large loops in the miRNA/miRNA* duplex; [2] up to five mismatched positions，only three of which are nucleotides in asymmetric bulges and miRNA* sequences (or at least approximate miRNA* sequences). If there is miRBase gff annotation file of miRNAs of corresponding species under miRBase directory, if the position of miRNAs identified by the program overlaps with that in gff file, the program will automatically select loose annotation standard for these sites due to the support of miRBase data (Users can delete gff file if need to use strict standards for all data)). Not strict miRNA annotation model, which is suitable for annotating newly generated miRNAs and conserved miRNAs together, require standard hairpin structure but the requirements for miRNA* sequences cannot be very strict (Default value: Strict annotation model).

(10) Check genome data:

Check the base data of the genome. If a chromosome has non-standard base data, the data of the chromosome will be screened out. (If choosing not to check the genome data, you must be very careful, as some non-standard base letters may affect the operation of the program! In addition, it's better to run the SRICATs program from the command line. If there is non-standard base data, the detailed information will be displayed) (Default value: Check genome data).

(11) Picture format:

Choose the format of miRNA secondary structure picture. Our graphical interface only displays images in PNG format, other formats of images are only generated to meet the needs of users (Default value: PNG)！

Intelligent prompts will be displayed via mousing over the corresponding regions.

**4.3. Identify miRNAs**

To analysis data, you can click “Analysis” menu and choose “Plant small data analysis”, then the program will show analysis process and results (see Figure 7).


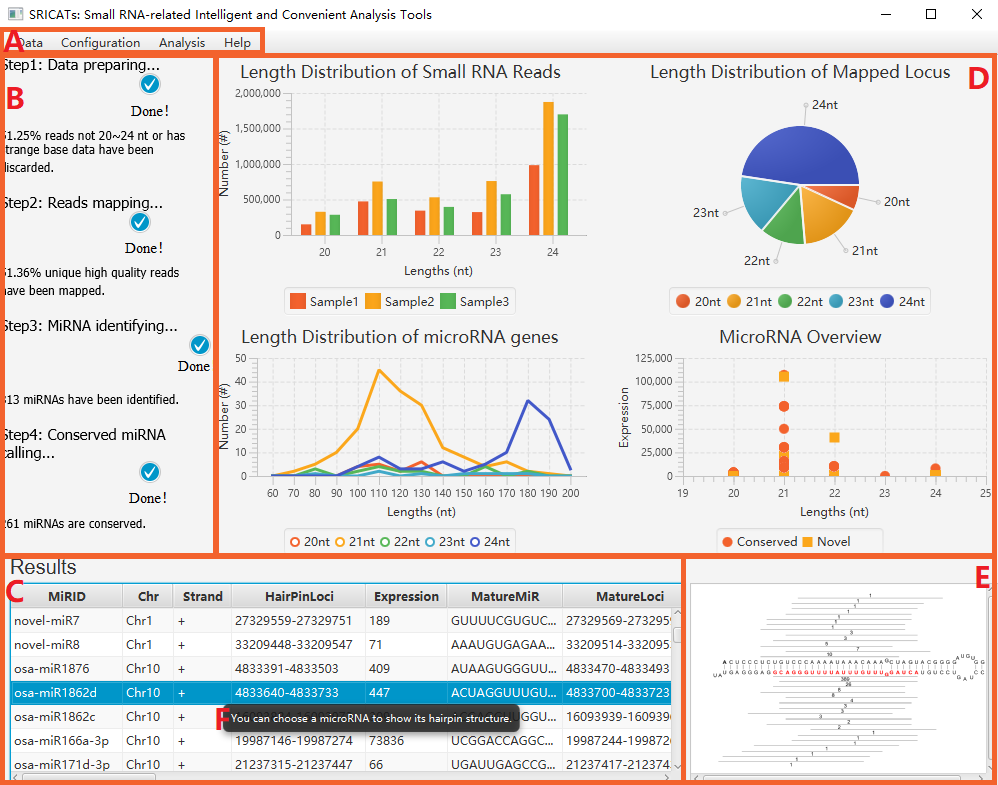


**Figure 7. Screenshot of SRICATs.** (A) Menu bar. (B) Progress panel showing task progress. (C) Results panel showing attributes of annotated miRNAs. (D) Statistical charts panel displaying some basic features of the uploaded dataset and results. (E) Graph panel displaying secondary structure of selected miRNA precursors. (F) Intelligent prompts will be displayed via mousing over the corresponding regions.

**4.4. Identify miRNA families**

SRICATs could identify miRNA families by comparing the outputs of the program to miRBase (7) by adding gff file （File name changed to knownMiR.gff3） of miRNA annotation of corresponding species downloaded from miRBase to miRBase directory of SRICATs package (Create a directory named miRBase under the directory where the SRICATs package is located).

**4.5. Help**

To see help information, you can click “Help” menu, then the help information will be displayed.

**4.6. Save results**

After analysis, you can click “Data” menu and choose “Save results” (see Figure 8), then click “ok” button (see Figure 9), pictures of miRNAs will be saved in tmp fold.


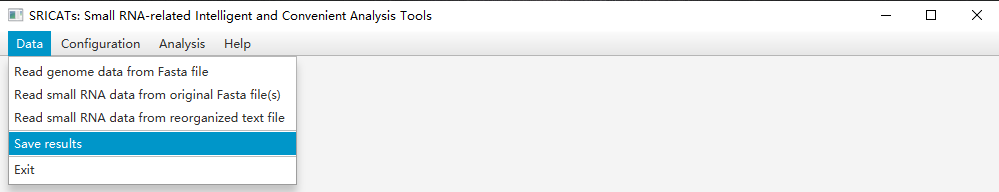


**Figure 8. Save results**


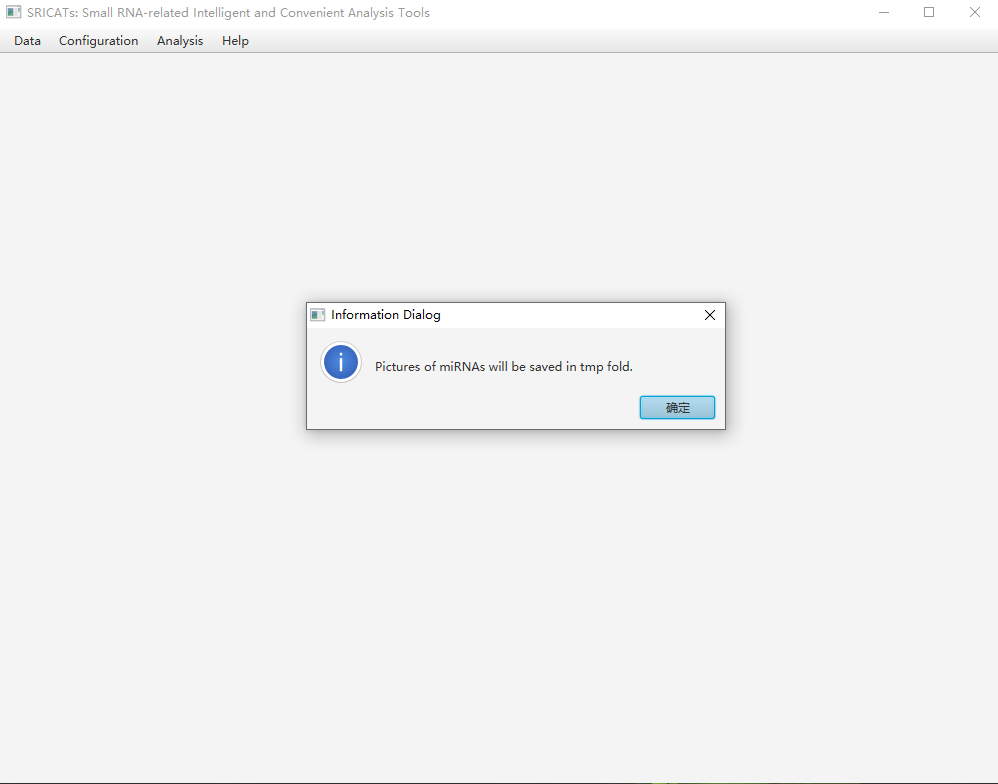


**Figure 9. Save results**

**4.7. Exit**

After analysis, you can click “Data” menu and choose “Exit”, the results will be saved in "results.txt" file.

1. **Example**

The test data of *Arabidopsis thaliana* genome can be downloaded from https://www.arabidopsis.org/.

The test data of *Arabidopsis thaliana* small RNA can be downloaded from https://www.ncbi.nlm.nih.gov/geo/query/acc.cgi?acc=GSM2094927.

1. **Addresses and Contacts**

Address: College of Information Engineering, Guizhou University of Traditional Chinese Medicine, Guizhou 550025, China.

E-mail: zhangyun016@gzy.edu.cn.

**7. Reference**

1. M. Li, B. Yu, Recent advances in the regulation of plant miRNA biogenesis. *RNA Biol* 10.1080/15476286.2021.1899491, 1-10 (2021).

2. B. C. Meyers *et al.*, Criteria for annotation of plant MicroRNAs. *Plant Cell* **20**, 3186-3190 (2008).

3. M. J. Axtell, Classification and comparison of small RNAs from plants. *Annu Rev Plant Biol* **64**, 137-159 (2013).

4. M. J. Axtell, B. C. Meyers, Revisiting Criteria for Plant MicroRNA Annotation in the Era of Big Data. *Plant Cell* **30**, 272-284 (2018).

5. G. Eraslan, Z. Avsec, J. Gagneur, F. J. Theis, Deep learning: new computational modelling techniques for genomics. *Nat Rev Genet* **20**, 389-403 (2019).

6. Y. Zhang, W. K. Jiang, L.Z. Gao, Evolution of microRNA genes in *Oryza sativa* and *Arabidopsis thaliana*: an updata of the inverted duplication model. PLos One 6, e28073 (2011).

7. A. Kozomara, M. Birgaoanu, S. Griffiths-Jones, miRBase: from microRNA sequences to function. *Nucleic Acids Res* **47**, D155-D162 (2019).
