# Supplementary material for: Identification of plant microRNAs using convolutional neural network
Source: Front Plant Sci. 2024 Mar 19;15:1330854. doi: 10.3389/fpls.2024.1330854 (PMC10985208; doi:10.3389/fpls.2024.1330854)
Supplement: Supplementary file 3 [file Table_1.docx]

**Table S1. CNN architectures and hyperparameters tested.**

| CNN architectures and hyperparameters | Value | Accuracy | Precision | Recall | F1-score |
| --- | --- | --- | --- | --- | --- |
| Number of convolution layers and pooling layers (The structure of the third layer is the same as that of the second layer) | 3 | 92.28% | 85.93% | 100.00% | 92.43% |
| Filter size of first convolution layer | 3*3 | 94.31% | 89.23% | 100.00% | 94.31% |
|  | 4*4 | 94.31% | 89.23% | 100.00% | 94.31% |
|  | 5*5 | 92.28% | 85.93% | 100.00% | 92.43% |
| Number of filters in first convolution layer | 30 | 89.02% | 81.12% | 100.00% | 89.58% |
|  | 40 | 94.72% | 89.92% | 100.00% | 94.69% |
|  | 50 | 94.31% | 89.23% | 100.00% | 94.31% |
|  | 60 | 93.90% | 88.55% | 100.00% | 93.93% |
|  | 70 | 92.28% | 85.93% | 100.00% | 92.43% |
|  | 80 | 92.68% | 86.57% | 100.00% | 92.80% |
|  | 90 | 93.50% | 87.88% | 100.00% | 93.55% |
|  | 100 | 94.31% | 89.23% | 100.00% | 94.31% |
|  | 300 | 94.31% | 89.23% | 100.00% | 94.31% |
|  | 500 | 91.06% | 84.06% | 100.00% | 91.34% |
| Activation function in first convolution layer | Identity | 97.56% | 95.08% | 100.00% | 97.48% |
|  | Tanh | 95.53% | 91.34% | 100.00% | 95.47% |
|  | Sigmoid | 69.92% | 61.05% | 100.00% | 75.82% |
| Filter size of second convolution layer | 3*3 | 91.06% | 84.06% | 100.00% | 91.34% |
|  | 4*4 | 95.93% | 92.06% | 100.00% | 95.87% |
|  | 5*5 | 92.68% | 86.57% | 100.00% | 92.80% |
| Number of filters in second convolution layer | 20 | 94.31% | 89.23% | 100.00% | 94.31% |
|  | 30 | 90.65% | 83.45% | 100.00% | 90.98% |
|  | 40 | 93.50% | 87.88% | 100.00% | 93.55% |
|  | 50 | 96.75% | 93.55% | 100.00% | 96.67% |
|  | 70 | 91.46% | 84.67% | 100.00% | 91.70% |
|  | 80 | 92.68% | 86.57% | 100.00% | 92.80% |
|  | 90 | 91.46% | 84.67% | 100.00% | 91.70% |
|  | 100 | 95.12% | 90.62% | 100.00% | 95.08% |
|  | 300 | 91.87% | 85.29% | 100.00% | 92.06% |
|  | 500 | 92.68% | 86.57% | 100.00% | 92.80% |
| Activation function in second convolution layer | Identity | 94.72% | 89.92% | 100.00% | 94.69% |
|  | Tanh | 93.09% | 87.22% | 100.00% | 93.17% |
|  | Sigmoid | 82.11% | 75.35% | 92.24% | 82.95% |
| Stride for filters | 2*2 | 91.46% | 84.67% | 100.00% | 91.70% |
| Using padding | Yes | 94.31% | 89.23% | 100.00% | 94.31% |
| Pooling method | Average | 92.68% | 86.57% | 100.00% | 92.80% |
| Filter size of pooling layer | 3*3 | 90.24% | 82.86% | 100.00% | 90.62% |
| Number of units in fully connected layer | 100 | 94.31% | 89.23% | 100.00% | 94.31% |
|  | 300 | 94.72% | 89.92% | 100.00% | 94.69% |
|  | 700 | 92.68% | 86.57% | 100.00% | 92.80% |
|  | 1000 | 96.34% | 92.80% | 100.00% | 96.27% |
| Activation function in fully connected layer | ReLU | 91.87% | 85.29% | 100.00% | 92.06% |
|  | Tanh | 96.75% | 95.00% | 98.28% | 96.61% |
|  | Sigmoid | 96.34% | 92.80% | 100.00% | 96.27% |
| Activation function in output layer | Sigmoid | 52.03% | 0.00% | 0.00% | 0.00% |
| Regularization technique | L1 | 95.93% | 92.06% | 100.00% | 95.87% |
|  | L2 | 95.53% | 91.34% | 100.00% | 95.47% |
| Dropout coefficient | 0.5 | 76.42% | 66.67% | 100.00% | 80.00% |
|  | 0.6 | 93.09% | 87.22% | 100.00% | 93.17% |
|  | 0.7 | 89.43% | 81.69% | 100.00% | 89.92% |
|  | 0.8 | 95.93% | 92.06% | 100.00% | 95.87% |
|  | 1.0 | 95.93% | 92.06% | 100.00% | 95.87% |
| Optimization Algorithm | Adam | 95.93% | 92.06% | 100.00% | 95.87% |
|  | AdaGrad | 77.24% | 68.75% | 94.83% | 79.71% |
|  | RMSProp | 47.15% | 47.15% | 100.00% | 64.09% |
|  | SGD | 71.95% | 64.07% | 92.24% | 75.62% |
| Loss function for classification | Hinge | 96.34% | 92.80% | 100.00% | 96.27% |
| Weight Initialization Strategies | ReLU | 96.75% | 93.55% | 100.00% | 96.67% |
