# Supplementary material for: Identification of plant microRNAs using convolutional neural network
Source: Front Plant Sci. 2024 Mar 19;15:1330854. doi: 10.3389/fpls.2024.1330854 (PMC10985208; doi:10.3389/fpls.2024.1330854)
Supplement: Supplementary file 4 [file Table_2.docx]

Table S2. The number of miRNAs identified by SRICATS, UEA sRNA workbench and miDeep-P2.

| Species | SRICATs | UEA sRNA workbench | miRDeep-P2 |
| --- | --- | --- | --- |
| *Arabidopsis thaliana* | 94 | 432 | 187 |
| *Oryza sativa* | 90 | 392 | 35 |
| *Sorghum bicolor* | 200 | 756 | 61 |
| *Chlamydomonas reinhardtii* | 64 | 84 | 58 |
| *Physcomitrella patens* | 100 | 93 | 67 |
| Total | 548 | 1757 | 408 |
| Average | 110 | 351 | 82 |
